# Postpartum Depressive Symptoms and Their Long-Term Association with Anxiety and Depression in Women: Findings from the Rhea Study in Crete, Greece

**DOI:** 10.3390/healthcare14060745

**Published:** 2026-03-16

**Authors:** Katerina Koutra, Chrysi Mouatsou, Katerina Margetaki, Georgios Mavroeides, Lida Chatzi

**Affiliations:** 1 Department of Psychology, School of Social Sciences, University of Crete, 741 00 Rethymno, Greecemavroeidesg@uoc.gr (G.M.); 2 Clinic of Ρreventive and Social Medicine, Department of Social Medicine, Faculty of Medicine, University of Crete, 700 13 Heraklion, Greece; 3 Department of Preventive Medicine, Keck School of Medicine, University of Southern California, Los Angeles, CA 90033, USA

**Keywords:** depressive symptoms, mental health, postpartum period, trait anxiety, women

## Abstract

**Highlights:**

**What are the main findings?**

- Postpartum depressive symptoms are associated with higher levels of depression and anxiety symptoms 11 and 15 years after childbirth.
- The link between postpartum depressive symptoms and anxiety strengthens over time, with a stronger association observed at the 15-year follow-up.

**What are the implications of the main findings?**

- Early postpartum depressive symptoms are associated with an increased likelihood of elevated depressive and anxiety symptoms later in life.
- There is a need for early screening and long-term support for mothers to mitigate the risk of enduring psychological difficulties.

**Abstract:**

**Background/Objectives**: Postpartum depression affects 10–20% of women and may have long-term consequences for mental health. This study examines the association between postpartum depressive symptoms and women’s depression and anxiety symptoms 11 and 15 years after childbirth. **Methods:** Data were drawn from the Rhea Mother–Child Cohort in Crete, Greece. A total of 1079 women completed the Edinburgh Postnatal Depression Scale (EPDS) at approximately 8–10 weeks postpartum. Of these, 516 participated in follow-up assessments at 11 and 15 years, which included measures of anxiety (State-Trait Anxiety Inventory, Trait version [STAI-Trait]) and depressive symptoms (Beck Depression Inventory [BDI]). Multivariable linear mixed-effects models were used to assess the relationship between postpartum depressive symptoms (EPDS) and later anxiety (STAI-Trait) and depression (BDI) outcomes, adjusting for sociodemographic and family-related characteristics and psychosocial factors. **Results:** Higher postpartum EPDS scores were associated with greater anxiety (STAI-Trait) and depressive symptoms (BDI) across follow-up assessments. Associations remained significant after adjusting for maternal baseline characteristics and follow-up factors. An interaction with time suggested that the association between postpartum depressive symptoms and anxiety levels strengthened over time, with a stronger association at the 15-year follow-up, indicating a higher long-term mental health burden. **Conclusions:** Postpartum depressive symptoms are associated with higher levels of depressive and anxiety symptoms 11 and 15 years after childbirth. The association with depression appeared relatively consistent across follow-up assessments, while the relationship with anxiety was stronger at the 15-year follow-up. These findings suggest that postpartum depression is an early marker of long-term vulnerability to mood and anxiety disorders, highlighting the importance of early screening, intervention, and long-term mental health support for mothers to reduce the risk of enduring psychological difficulties.

## 1. Introduction

Perinatal maternal mental health, encompassing women’s psychological well-being during pregnancy and the postpartum period, is a critical determinant of both short- and long-term outcomes for mothers and their children [1]. The perinatal period involves profound physical, emotional, and social changes and is increasingly recognized as a pivotal window for mental health assessment and intervention [2]. Among perinatal mental health conditions, postpartum depression (PPD) is particularly prevalent. A systematic review of 58 studies (N = 37,294) reported a PPD incidence of 12% and a prevalence of 17% [3]. Maternal PPD increases the risk of both long-term maternal mental health problems and child emotional and behavioral difficulties [4], with effects extending from early childhood through adolescence [5].

PPD symptoms often extend beyond the early postnatal period, following a persistent or recurrent course and increasing the risk of subsequent affective disorders later in life [6]. According to the systematic review by Vliegen et al. [7], although most women recover from PPD, approximately 23–49% (median 38%) of women with PPD continue to experience chronic or relapsing depressive symptoms over time. Longitudinal research further demonstrates that PPD symptomatology can have enduring consequences for maternal mental health, persisting for up to a decade or more after childbirth. Specifically, findings from the Avon Longitudinal Study of Parents and Children (ALSPAC) revealed that mothers with severe and persistent PPD symptoms exhibited significantly elevated levels of depression even 11 years postpartum [8]. Moreover, symptom severity appears to play a critical role in recovery trajectories, with more severe PPD associated with a slower decline in depressive symptoms over time [9]. In the Southeast Sweden Birth Cohort, Agnafors et al. [10] found that scoring above the EPDS cutoff at three months postpartum was associated with an increased risk of elevated depressive symptoms even 25 years later. Similarly, Lee et al. [11] reported that women with a history of PPD had a significantly higher cumulative risk of suicide at 5, 10, and 15 years postpartum compared to mothers without PPD. Finally, Bloch et al. [12] found that 42.5% of women with a history of PPD developed a new depressive episode within five years, compared with only 3.7% of women without PPD. Furthermore, women with prior PPD demonstrated higher overall psychopathology and poorer functional outcomes at follow-up.

Few studies have explored depressive symptoms in women 15 years after childbirth, making the present study, conducted in Greece, a valuable contribution to understanding long-term maternal mental health in Southern European populations. To our knowledge, this is the first study to assess both depressive and anxiety symptoms in the years following childbirth, providing a more comprehensive understanding of long-term psychological outcomes. By providing longitudinal data from a Greek cohort, this research addresses an important gap in the literature and provides insight into the long-term associations between early PPD symptoms and later maternal mental health. According to our hypothesis, women who experience PPD symptoms are more likely to report elevated depressive and anxiety symptoms later in life. This study examines these associations within a Mediterranean context to improve understanding of maternal mental health trajectories and to inform strategies for early identification, support, and prevention of long-term mental health difficulties.

## 2. Materials and Methods

### 2.1. Participants

This study is nested within the Rhea Study, a prospective mother–child cohort investigating environmental, dietary, and psychosocial determinants of health among pregnant women and their children in Heraklion, Crete, Greece. Female residents, including both Greek nationals and immigrants who became pregnant within a defined 12-month period beginning in February 2007, were invited to participate. Recruitment occurred at four maternity clinics in Heraklion, where eligible women—aged 16 years or older and proficient in Greek—were approached during their first major ultrasound examination, conducted before the 15th week of gestation. Trained nurses and midwives provided detailed information about the study and obtained informed consent. Participants were subsequently followed during the third trimester, at delivery, and at 8–10 weeks postpartum. Data collection involved face-to-face structured interviews, self-administered questionnaires, and review of medical records, capturing extensive information on psychosocial, dietary, and environmental exposures during pregnancy and the postpartum period. Several follow-up assessments were conducted across key stages of child development, including infancy (9 and 18 months), early and middle childhood (4 and 6 years), preadolescence (11 years), and adolescence (15 years). Maternal mental health was assessed at the 11- and 15-year follow-ups.

**Figure 1:**
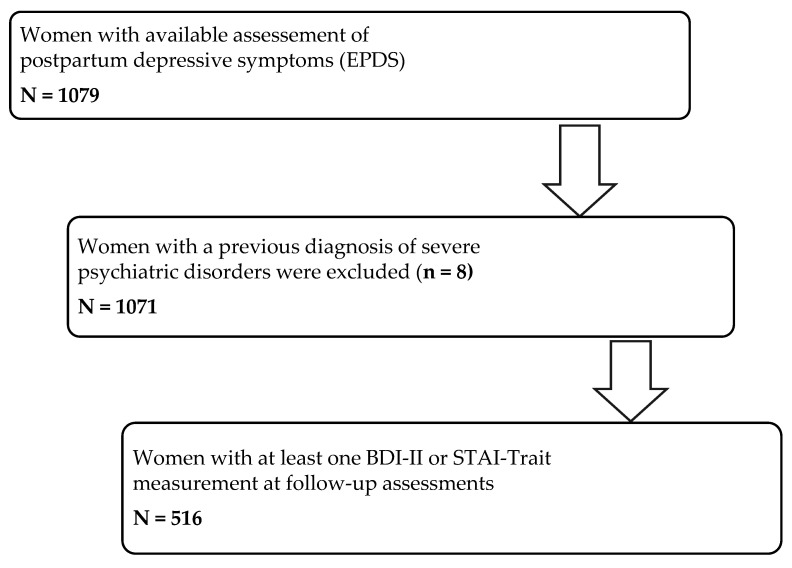
Flowchart of the study population. *Abbreviations:* BDI-II: Beck Depression Inventory-II; EPDS: Edinburgh Postnatal Depression Scale; STAI: State-Trait Anxiety Inventory.

Comprehensive details on the study population have been reported elsewhere [13]. The study initially recruited 1765 eligible women, of whom 1610 (91%) consented to participate, and 1388 (86%) were followed through delivery. A total of 1079 women completed the depressive symptoms assessment at 8–10 weeks postpartum. Eight women with a history of severe psychiatric disorders (e.g., schizophrenia and bipolar disorder) were excluded. Of the remaining 1071 women, 516 had data available for at least one follow-up assessment of depression or anxiety. Thus, the final analytic sample included 516 women with data from the postpartum period and at least one subsequent follow-up assessment (Figure 1).

### 2.2. Measures

#### 2.2.1. Maternal Sociodemographic Characteristics

Maternal sociodemographic characteristics were collected both at baseline and during follow-up assessments. At baseline, information was obtained on maternal age at delivery, country of origin, educational level, marital status, parity, working status, smoking status during pregnancy, and breastfeeding. At follow-up, updated sociodemographic information was collected, including maternal age at the 11- and 15-year assessments, family income and working status at both follow-up timepoints, as well as cohabitation status and number of children at the 15-year follow-up.

#### 2.2.2. Maternal Depressive Symptoms at the Postpartum Period

PPD symptoms were assessed with the *Edinburgh Postnatal Depression Scale* (EPDS) [14], a widely used 10-item self-report questionnaire designed for use during both the antenatal and postnatal periods. Items are rated on a 4-point Likert scale (0–3), yielding total scores ranging from 0 to 30, with higher scores reflecting more severe depressive symptomatology. The EPDS has demonstrated strong psychometric properties across perinatal populations [15,16] and has been validated for use in Greece [17,18]. In the present study, EPDS scores were analyzed both as a continuous measure and as a categorical indicator, with scores ≥ 13 denoting clinically significant depressive symptoms, consistent with established cut-offs [14].

#### 2.2.3. Maternal Depressive and Anxiety Symptoms at 11- and 15-Year Follow-Up

*Depressive symptoms* were assessed using the *Beck Depression Inventory-II* (BDI-II; [19]), which is a 21-item self-report questionnaire assessing core depressive symptoms, including sadness, anhedonia, self-criticism, fatigue, and disturbances in sleep and appetite, based on DSM-IV criteria (American Psychiatric Association, 1994) [20]. Items are scored on a 4-point scale (0–3), producing a total score between 0 and 63, with higher scores reflecting greater symptom severity. The BDI-II has been translated and validated for Greek populations [21].

*Trait anxiety* was measured using the trait subscale of the *State-Trait Anxiety Inventory* (STAI; [22]), which assesses a stable disposition toward anxiety. The subscale consists of 20 items rated on a 4-point scale from 1 (Almost never) to 4 (Almost always), with total scores ranging from 20 to 80; higher scores indicate higher trait anxiety. The STAI has been translated and adapted for use in the Greek population [23].

### 2.3. Procedure

Postnatal assessment at 8–10 weeks postpartum was conducted via telephone interviews by trained interviewers using the EPDS to evaluate symptoms of PPD. Maternal mental health was assessed longitudinally using validated self-administered questionnaires (STAI-Trait and BDI-II) at successive follow-ups. At each wave, participants received updated study information and were invited to complete the assessments either in person or via a secure digital platform. Data collection for the 11-year (June 2018–June 2019) and 15-year (August 2022–August 2023) follow-ups followed this standardized procedure. Among the 1268 eligible mothers, 947 (74.7%) were successfully reached, and 591 (62.4%) took part in the 15-year evaluation. After completing the assessment, participants were offered the opportunity to attend a counseling session with a psychologist from the Rhea team. Written informed consent was obtained from all participants. The study adhered to the principles of the Declaration of Helsinki and received ethical approval from the Ethics Committee of the University Hospital of Heraklion at baseline (reference number: 96/06.02.2007) and from the Research Ethics Committee of the University of Crete at the most recent follow-up (reference number: 43/16.03.2022), as part of the IntExt Trajectories project.

### 2.4. Statistical Analysis

Descriptive statistics were used to summarize the sample characteristics, exposure variables, and outcomes of interest. Continuous variables were presented as means with standard deviations (SD), while categorical variables were summarized as frequencies and percentages. The normality of continuous variables was assessed using the Shapiro–Wilk test, with non-parametric tests applied where appropriate. Bivariate associations with the psychometric scales (STAI-Trait and BDI) were analyzed using Mann–Whitney U tests or Kruskal–Wallis tests for categorical variables, and Spearman’s correlation coefficients for continuous variables.

To address missing data at the 11- and 15-year follow-up assessments, and to increase sample size and reduce bias, multiple imputation by chained equations (MICE) was employed. The imputation model included the exposure (EPDS), outcome variables (STAI-Trait and BDI at 11 and 15 years), and sociodemographic and clinical covariates. Twenty imputed datasets were generated, and results were combined using Rubin’s rules. The distributions of sociodemographic characteristics, exposure, and outcomes in the imputed datasets were similar to the observed data (Supplementary Table S1).

Effect estimates were obtained using linear mixed-effects models to examine the association between PPD symptoms (EPDS) and later anxiety (STAI-Trait) and depression (BDI) at 11 and 15 years after childbirth. Mixed models included a random intercept for each woman and a random slope for age. Potential confounders associated with the exposure or outcomes in bivariate analyses (*p* < 0.20), as well as a priori selected variables, were included in multivariable models. Collinearity was assessed using variance inflation factors (VIF < 10). Four models were constructed: Model 1 (crude) assessed the overall relationship between EPDS and mental health outcomes without considering time-related variations. Model 2 adjusted for baseline characteristics, including maternal age at delivery (years), maternal education at pregnancy (low <9 years/middle 9–12 years/high >12 years), parity (primiparous/multiparous), smoking status during pregnancy (never smoker/ex-smoker/current smoker), and marital status (married/other). Model 3 extends Model 2 by adding an interaction term between PPD and timepoint to examine whether the association between PPD and later mental health outcomes varies over time. Model 4 was fully adjusted, incorporating confounders of model 2 and additional factors, including family income at 11 and 15 years, maternal working status at 11 and 15 years, negative life events at 15 years, cohabitation status at 15 years, and number of children at 15 years.

All hypothesis testing was conducted at a significance level of 0.05 with a two-sided alternative hypothesis. Statistical analyses were performed using Stata software, version 13.0 (StataCorp, College Station, TX, USA).

## 3. Results

### 3.1. Descriptive Statistics

Table 1 presents the sociodemographic characteristics of women at baseline and at follow-up assessments. Women had a mean age of 30.1 (±4.6) years at delivery, 41.4 (±4.3), and 45.5 (±4.7) years at the 11-year and 15-year follow-up assessments, respectively. The majority were Greek (96.5%), had middle (50.6%) or high education (38.6%), and were married (90.7%) at baseline. Slightly more than half (53.9%) were multiparous at baseline. At the 11-year follow-up, most women were employed (74.3%), and 9.9% reported insufficient family income. At 15 years, a higher proportion of women were employed (86.6%), while a similar proportion (9.3%) reported insufficient income. Most women were living with the father of the child (88.1%), and about half of the families (52.4%) had two children at the 15-year follow-up.

The descriptive results of mental health assessments during the postpartum period and at follow-ups are presented in Table 2. During the postpartum period, 11.6% of women reported elevated depressive symptoms (EPDS ≥ 13). At later timepoints, fewer women reported increased depressive symptoms (BDI ≥ 17): 8.9% at 11 years and 7.5% at 15 years.

Non-response analyses indicated significant differences in baseline characteristics between participants (women with both postpartum and follow-up data, N = 516) and non-participants (women with only postpartum data, N = 563). Specifically, participating women were slightly older, were more likely to be Greek, and had higher educational attainment. Furthermore, they were more likely to be married, primiparous, employed, non-smokers, and to have breastfed their child, compared with women excluded from the analyses. In addition, participating women reported lower levels of depressive symptoms in the postpartum period (Supplementary Table S2).

### 3.2. Bivariate Associations Between Postpartum Depressive Symptoms and Mental Health in Later Life, as per Timepoint Assessment

The bivariate associations between PPD symptoms and women’s mental health outcomes later in life were positive and statistically significant (Table 3). Specifically, PPD symptoms were associated with higher depressive symptoms at 11 and 15 years (rho = 0.20, *p* = 0.001 and rho = 0.18, *p* < 0.001, respectively). The associations were slightly stronger for anxiety, with PPD symptoms linked to higher levels of anxiety at 11 and 15 years (rho = 0.24, *p* < 0.001 and rho = 0.28, *p* < 0.001, respectively).

### 3.3. Associations Between Postpartum Depressive Symptoms and Mental Health in Later Life Across Time: Linear Mixed-Effects Models

The results from the linear mixed-effects models are illustrated in Table 4. Higher PPD symptoms were associated with elevated levels of depressive and anxiety symptoms over time (crude models: beta = 0.32; 95% CI: 0.19–0.45 for depression; beta = 0.30; 95% CI: 0.19–0.41 for anxiety). These associations remained significant in models adjusted for baseline characteristics (beta = 0.31; 95% CI: 0.17–0.44 for depression; beta = 0.29; 95% CI: 0.18–0.40 for anxiety). Similar results were observed when PPD symptoms were treated as a categorical variable (EPDS ≥ 13), with elevated PPD symptoms linked to higher depressive symptoms and anxiety across time (beta = 3.65; 95% CI: 1.59–5.78 for depression; beta = 2.45; 95% CI: 0.84–4.07 for anxiety).

The inclusion of an interaction term between PPD symptoms and timepoint (Model 3) revealed significant variations over time for anxiety. The interaction was statistically significant (*p* = 0.004), with a stronger association between PPD and anxiety being observed at the 15-year follow-up assessment (11 years: beta = 0.19; 95% CI: 0.07–0.31; 15 years: beta = 0.44; 95% CI: 0.28–0.60). The results from models using categorical PPD symptoms were similar, with a statistically significant interaction with time (*p* = 0.032) and a significant relationship observed only at the 15-year follow-up (11 years: beta = 1.48; 95% CI: −0.13–3.10; 15 years: beta = 4.00; 95% CI: 1.56–6.44). For depression, no significant interactions with time emerged (*p* = 0.207 for continuous EPDS and *p* = 0.206 for categorical EPDS). However the association between PPD and future depressive symptoms was slightly stronger at 11 years both in the model using continuous EPDS (11 years: beta = 0.36; 95% CI: 0.18–0.53; 15 years: beta = 0.26; 95% CI: 0.13–0.40) and in the model using categorical EPDS (11 years: beta = 4.47; 95% CI: 1.82–7.11; 15 years: beta = 2.86; 95% CI: 0.65–5.06).

In models further adjusted for characteristics at both baseline and subsequent timepoints (Model 4), the associations across time remained significant, but they were slightly attenuated (Continuous EPDS; beta = 0.24, 95% CI; 0.12–0.36 for depression, beta = 0.25, 95% CI; 0.14–0.36 for anxiety, Categorical EPDS; beta = 2.80, 95% CI; 0.91–4.68 for depression, beta = 1.94, 95% CI; 0.33–3.56 for anxiety).

## 4. Discussion

The present study found that PPD symptoms are significantly associated with increased depressive and anxiety symptoms in women 11 and 15 years after childbirth. Specifically, higher levels of PPD symptoms were associated with greater long-term symptom burden across multiple analytical models, including those adjusted for maternal baseline characteristics and subsequent life course factors, indicating that the role of PPD is significant well beyond the perinatal period. The findings further suggest differential temporal patterns for depression and anxiety. While the association between PPD and depressive symptoms was generally consistent over time, the relationship with anxiety strengthened at the 15-year follow-up, highlighting anxiety as a potentially more persistent outcome of early postpartum distress. These patterns were observed regardless of whether PPD symptoms were treated as continuous or categorical measures, reinforcing the robustness of the findings.

Depressive symptoms in the postnatal period were consistently found to be associated with depressive symptoms in women’s later life, a finding supported by a substantial body of longitudinal research [7,8,10,12]. Collectively, these studies indicate that early manifestations of PPD are not merely transient for many women but often signal the onset of a persistent or recurrent trajectory of affective symptoms. Rather than reflecting a transient adjustment response to childbirth, early PPD symptoms appear to mark a subgroup of women with elevated vulnerability to symptom persistence or recurrence across the life course [24]. Prior studies have demonstrated heterogeneous symptom trajectories following childbirth, with a substantial proportion of affected women experiencing stable or escalating depressive symptoms over several years [7,10,12]. These ongoing patterns may reflect the interaction of biological susceptibility, ongoing psychosocial stressors, and gaps in the continuity of mental health care beyond the early postpartum period [9]. In the present study, depressive symptoms were assessed using different instruments across timepoints (EPDS postpartum and BDI-II at follow-up), reflecting the practical evolution of measurement tools in longitudinal research. Despite these differences, early PPD symptoms remained linked to elevated depressive symptoms later in life, supporting the notion that postpartum symptom assessment can provide valuable insight into long-term mental health trajectories.

Beyond its established link with long-term depressive outcomes, PPD symptomatology in our study was also found to be associated with subsequent anxiety symptoms. Previous research suggests that PPD symptoms are linked to early postpartum [25] and even sustained early postpartum anxiety (i.e., up to eight weeks after birth) [26]. However, the long-term trajectory of this relationship has been understudied. To our knowledge, the present study is among the first to examine the association between PPD symptoms and anxiety symptoms across an extended follow-up period, addressing an important gap in maternal mental health research. It is important to note that anxiety outcomes in this study were assessed using a trait-based measure (STAI-Trait), which captures relatively stable dispositional characteristics rather than episodic symptoms. Therefore, the observed associations likely reflect broader vulnerability or predisposition to anxiety rather than direct downstream effects of PPD symptoms. Trait anxiety, defined as a general tendency to experience anxiety across various situations over time, may be particularly relevant in understanding how PPD contributes to long-term anxiety outcomes [22]. Overall, these results underscore the importance of early identification, supportive intervention, and long-term monitoring to promote maternal mental health and reinforce the need for further longitudinal studies to clarify the mechanisms linking PPD symptoms with later anxiety outcomes.

The observed association of higher PPD symptoms with anxiety and depressive symptoms over time likely reflects a convergence of biological, psychological, and social mechanisms. Biologically, PPD has been linked to dysregulation of stress-response systems, particularly the hypothalamic–pituitary–adrenal (HPA) axis, as well as alterations in reproductive and stress-related hormones, with persistent disruptions in cortisol and neurotransmitter systems (serotonin, dopamine) increasing vulnerability to later affective disorders [27,28,29]. Psychologically, early PPD symptoms may foster maladaptive cognitive and emotional patterns, including rumination, negative mood bias, heightened stress reactivity, and impaired coping, which can perpetuate both depressive and anxiety symptoms over time [30,31]. Social and behavioral pathways further contribute, as PPD often leads to fatigue, emotional instability, reduced motivation, and withdrawal from social and health-promoting activities, potentially disrupting caregiving routines, straining partner relationships, and weakening social support networks, thereby amplifying psychological distress [30,31]. Collectively, these interrelated mechanisms suggest that PPD may act as an early stressor, initiating a cascade of biological vulnerability and psychosocial strain that contributes to the enduring burden of anxiety and depression, highlighting the importance of early identification and intervention to mitigate long-term mental health consequences.

The findings of the present study further indicate variation in the course of depression and anxiety following PPD. While the association between PPD and subsequent depressive symptoms remained relatively stable over time, the link with anxiety became more pronounced at the 15-year follow-up. This suggests that anxiety may represent a particularly enduring outcome of early postpartum distress, potentially emerging or intensifying long after the initial depressive episode. These patterns are consistent with evidence that depression and anxiety, while often comorbid, follow partially distinct longitudinal trajectories [32,33]. Whereas depressive symptoms may show more immediate and persistent continuity after PPD, anxiety symptoms could be influenced by cumulative stress, life events, or ongoing caregiving challenges, which may amplify vulnerability over time. PPD may confer long-term susceptibility to anxiety through shared biological and psychological pathways, including dysregulation of the HPA axis, heightened stress reactivity, and maladaptive cognitive-emotional processing [31]. Moreover, early PPD symptoms could act as a sensitizing factor, lowering the threshold for future anxiety disorders in response to later stressors. Finally, maternal anxiety may be under-recognized in the postpartum period, leading to insufficient intervention and allowing symptoms to persist or escalate across the life course.

The strengths of this study include its large, population-based sample of women, well-characterized participants, and longitudinal design. The 15-year follow-up, incorporating multiple data collection points, offers a valuable opportunity to examine associations of PPD symptoms with depressive and anxiety symptoms in women’s later life. While the study’s findings offer important insights, repeated assessments using the same psychometric instruments (BDI and STAI-Trait) at both the 11- and 15-year follow-ups would have further strengthened the robustness and consistency of the results.

Nevertheless, several limitations should be considered when interpreting these findings. Although the longitudinal design is a major strength, substantial attrition from the original cohort and systematic differences between participants and non-participants— where participants exhibited lower PPD symptoms and more favorable sociodemographic characteristics than non-participants —may have introduced selection bias; therefore, despite the use of multiple imputation, the long-term associations observed should be interpreted with caution and may have limited generalizability. Analyses included only women with postpartum data and at least one subsequent follow-up, which reduced the sample size and may have limited the ability to detect subtle effects, warranting cautious interpretation of effect sizes. Attrition was particularly pronounced at the 11-year follow-up, leading to less available data at that timepoint, although the large baseline cohort still enabled repeated assessments in a substantial subgroup.

A key limitation of this study is the missing data for depression and anxiety at year 11, which is likely not random, with higher scores associated with missingness. This introduces potential information bias, as the absence of data may lead to an underestimation of depression and anxiety levels at the 11-year follow-up. Depressive symptoms were assessed using different validated instruments across timepoints (EPDS postpartum and BDI-II at 11 and 15 years), which introduces potential measurement heterogeneity and limits direct comparability of symptom severity over time; therefore, findings related to symptom trajectories should be interpreted with caution. In addition, anxiety and depressive symptom levels at long-term follow-up may have been influenced by concurrent life stressors that were not fully captured in the analyses, potentially contributing to elevated symptom scores independent of PPD symptoms. Furthermore, information on maternal depression and anxiety prior to pregnancy was not available, which may have influenced both PPD symptoms and long-term outcomes. Medical comorbidities and long-term medication use were not systematically collected, and these factors may have influenced long-term mental health outcomes. Another key limitation is the inability to establish causality due to the observational nature of the study. While long-term associations between PPD symptoms and later mental health outcomes were observed, we cannot infer causality. The absence of data on symptom course, recurrence, treatment, or major life events during the follow-up period limits our ability to assess the sustained impact of PPD and potential confounding factors.

An additional limitation is the absence of intermediate assessments of maternal mental health between the early postpartum period and the long-term follow-up assessments. Consequently, the study design does not allow differentiation between persistent vulnerability, remission followed by later recurrence, chronic symptom trajectories, or independent onset of depressive or anxiety symptoms later in life. Furthermore, potential influences occurring during the intervening years, such as treatment, medication use, or cumulative life stressors, could not be examined. Therefore, the observed associations should be interpreted with caution, as the available data do not permit conclusions regarding symptom trajectories or mechanisms linking early PPD symptoms with later mental health outcomes. Finally, the cohort consists predominantly of Greek women from a single geographic region, with high rates of marriage, employment, and breastfeeding, which enhances internal validity but limits the generalizability of findings to more socioeconomically diverse populations or non-Mediterranean contexts; therefore, caution is warranted when extrapolating these results to broader maternal mental health policy and practice.

Given the study’s limitations and the substantial temporal gap between postpartum assessment and later follow-ups, these findings should be interpreted with caution. PPD symptoms, assessed around 8–10 weeks after childbirth, may not directly lead to long-term mental health difficulties but rather act as a non-specific marker of vulnerability to later depressive or anxiety symptoms. This suggests that women who experience PPD symptoms may have pre-existing risk factors—such as genetic predisposition or prior mental health history—that increase their likelihood of developing similar symptoms or other mental health challenges over time. Thus, the observed association between PPD and later mental health outcomes may reflect broader vulnerability rather than a sustained influence of PPD itself.

## 5. Conclusions

The present study provides robust evidence that PPD symptoms are associated with elevated depressive and anxiety symptoms 11 and 15 years after childbirth. While the association with depression remained stable over time, the link to anxiety was more pronounced at the 15-year follow-up. Given that depressive and anxiety symptoms share common underlying mechanisms, including emotional dysregulation, cognitive vulnerability, and heightened stress reactivity, PPD may represent an early marker of sustained vulnerability to both mood and anxiety disorders later in life. These findings emphasize the importance of early identification and intervention for PPD symptoms, not only to improve short-term maternal well-being but also to mitigate long-term mental health consequences across the life course. Although this study cannot capture symptom trajectories or intervening influences across the life course, the observed long-term associations highlight the relevance of early postpartum mental health and the need for future studies with repeated assessments to better understand maternal mental health across time.

## 6. Implications for Research and Clinical Practice

Maternal depressive symptoms in the postpartum period have important implications for women’s long-term psychological well-being. The findings of the present study highlight the need to strengthen both empirical research and clinical care in maternal mental health. Future studies should prioritize longitudinal designs to clarify the course of PPD and its relationship with later depressive and anxiety outcomes, while also identifying the biological, psychological, and social mechanisms that contribute to these patterns. Additionally, research should evaluate the effectiveness of screening tools, interventions, and follow-up strategies across diverse populations. These findings further emphasize the importance of extended monitoring beyond the early postpartum period, as women with elevated depressive symptoms may develop clinically significant anxiety even years after childbirth.

From a clinical perspective, effective management of PPD is essential for preventing long-term maternal mental health problems. Integrating PPD screening into routine obstetric and pediatric visits can facilitate early identification of women at risk for ongoing emotional difficulties [34]. Clinicians should be equipped to recognize early symptoms and provide timely psychosocial care, supported by structured follow-up for women with elevated depressive symptoms after childbirth [35]. Expanding access to mental health services, peer support, and community-based resources may further mitigate the long-term effects of PPD and improve outcomes for mothers, families, and children. Importantly, the findings highlight the need for continued assessment beyond the early postpartum period, with attention to both depressive and anxiety symptoms. Care plans should address anxiety directly, even when depressive symptoms diminish, to reduce the risk of persistent anxiety disorders.

Given the distinct trajectories of depression and anxiety following PPD, individualized and sustained prevention strategies are warranted. Psychotherapy, particularly cognitive-behavioral therapy and interpersonal therapy, should be prioritized as first-line treatments, as both have demonstrated efficacy in reducing depressive symptoms and enhancing maternal well-being [36,37]. In addition, proactive, theory-based interventions, including psychoeducational and mindfulness programs, show promising potential for improving outcomes during this critical period [38]. At the population and public health level, these findings underscore the importance of integrating maternal mental health services into standard healthcare systems, promoting continuity of care across the life course, and investing in long-term support strategies to optimize maternal and family well-being.

## Figures and Tables

**Table 1:** Sociodemographic characteristics of women at baseline and at follow-up assessments (N = 516).

Maternal Characteristics at Baseline	N (%) or Mean (SD)	Maternal Characteristics at Follow-Up Assessments	N (%) or Mean (SD)
**Age**		**Age**	
At delivery (N = 513)	30.1 (4.6)	At the 11-year follow-up (N = 274)	41.4 (4.3)
**Origin**		At the 15-year follow-up (N = 462)	45.5 (4.7)
Greek	497 (96.5)	**Family income at 11 years**	
Other	18 (3.5)	Sufficient	150 (55.1)
**Education** (pregnancy)		Sufficient with problems	95 (34.9)
Low (<9 years)	55 (10.8)	Insufficient	27 (9.9)
Middle (9–12 years)	258 (50.6)	**Family income at 15 years**	
High (>12 years)	197 (38.6)	Sufficient	221 (47.8)
**Marital status** (pregnancy)		Sufficient with problems	198 (42.9)
Married	458 (90.7)	Insufficient	43 (9.3)
Other	47 (9.3)	**Working status at 11 years**	
**Parity**		Not working	70 (25.7)
Primiparous	229 (46.1)	Working	202 (74.3)
Multiparous	268 (53.9)	**Working status at 15 years**	
**Working status** (pregnancy)		Not working	62 (13.4)
Not working	109 (21.8)	Working	400 (86.6)
Working	391 (78.2)	**Cohabitation status at 15 years**	
**Smoking status** (pregnancy)		Living with the father of the child	406 (88.1)
Never smoker	321 (64.2)	Other	55 (11.9)
Ex-smoker (quit during pregnancy)	87 (17.4)	**Number of children at 15 years**	
Current smoker	92 (18.4)	1 child	43 (9.3)
**Breastfeeding**		2 children	242 (52.4)
No	62 (12.0)	3 children	132 (28.6)
Yes	454 (88.0)	4 or more children	45 (9.7)
		**Negative life events at 15 years**	
		No	133 (29.8)
		Yes	314 (70.2)

**Table 2:** Descriptive characteristics of women’s depressive and anxiety symptoms at baseline and at follow-up assessments.

	Ν	Mean (SD)
**Depressive symptoms**		
Postpartum period (EPDS)	516	6.3 (4.9)
11-year follow-up (BDI-II)	258	7.4 (6.5)
15-year follow-up (BDI-II)	455	7.1 (6.1)
**Anxiety**		
11-year follow-up (STAI-Trait)	252	42.5 (4.7)
15-year follow-up (STAI-Trait)	461	38.6 (8.1)
	**Ν**	**%**
**Elevated depressive symptoms**		
Postpartum period (EPDS ≥ 13)	60	11.6
11-year follow-up (BDI ≥ 17)	23	8.9
15-year follow-up (BDI ≥ 17)	34	7.5

*Abbreviations:* BDI-II: Beck Depression Inventory-II; EPDS: Edinburgh Postnatal Depression Scale; STAI: State-Trait Anxiety Inventory.

**Table 3:** Bivariate associations between postpartum depressive symptoms and depressive and anxiety symptoms at follow-up assessments.

	N	Spearman’s Rho	*p*-Value
**Postpartum depressive symptoms (EPDS)**			
Depressive symptoms at 11-year follow-up (BDI-II)	258	0.20	**0.001**
Depressive symptoms at 15-year follow-up (BDI-II)	455	0.18	**<0.001**
Anxiety at 11-year follow-up (STAI-Trait)	252	0.24	**<0.001**
Anxiety at 15-year follow-up (STAI-Trait)	461	0.28	**<0.001**

*Abbreviations:* BDI-II: Beck Depression Inventory-II; EPDS: Edinburgh Postnatal Depression Scale; STAI: State-Trait Anxiety Inventory. Bold font indicates *p* < 0.05. *Footnote:* The variation in participant numbers between the 11-year and 15-year follow-up waves is due to differences in response rates rather than gradual attrition.

**Table 4:** Associations across time between postpartum depressive symptoms and depressive and anxiety symptoms at follow-up assessments, estimated using linear mixed-effects models (N = 516).

	Postpartum Depressive Symptoms (Continuous EPDS)		Postpartum Depressive Symptoms (Categorical EPDS ≥ 13)	
	Depression (BDI-II)	Anxiety (STAI-Trait)	Depression (BDI-II)	Anxiety (STAI-Trait)
	Beta (95% CI)	Beta (95% CI)	Beta (95% CI)	Beta (95% CI)
**Model 1**				
Across time	**0.32 (0.19–0.45)**	**0.30 (0.19–0.41)**	**3.87 (1.77–5.98)**	**2.57 (0.98–4.16)**
**Model 2**				
Across time	**0.31 (0.17–0.44)**	**0.29 (0.18–0.40)**	**3.65 (1.59–5.78)**	**2.45 (0.84–4.07)**
**Model 3**				
At 11-year follow-up	**0.36 (0.18–0.53)**	**0.19 (0.07–0.31)**	**4.47 (1.82–7.11)**	1.48 (–0.13–3.10)
At 15-year follow-up	**0.26 (0.13–0.40)**	**0.44 (0.28–0.60)**	**2.86 (0.65–5.06)**	**4.00 (1.56–6.44)**
p-interaction with time	0.207	**0.004**	0.206	**0.032**
**Model 4**				
Across time	**0.24 (0.12–0.36)**	**0.25 (0.14–0.36)**	**2.80 (0.91–4.68)**	**1.94 (0.33–3.56)**

*Abbreviations:* BDI-II: Beck Depression Inventory-II; EPDS: Edinburgh Postnatal Depression Scale; STAI-Trait: State-Trait Anxiety Inventory-Trait. Bold font indicates *p* < 0.05. **Model 1:** Crude model. **Model 2** (adjusted for baseline characteristics): maternal age at delivery (years), maternal education at pregnancy (low: <9 years/middle: 9–12 years/high: >12 years), parity (primiparous/multiparous), smoking status during pregnancy (never smoker/ex-smoker/current smoker), and marital status (married/other). **Model 3:** Model 2 including an exposure ∗ timepoint interaction term. **Model 4:** Model 2 adjusted for family income at 11 and 15 years, maternal working status at 11 and 15 years, negative life events at 15 years, cohabitation status at 15 years, and number of children at 15 years.

## Data Availability

The data presented in this study are available on request from the corresponding author. The data are not publicly available due to privacy and ethical restrictions.

## Supplementary Materials

The following supporting information can be downloaded at https://www.mdpi.com/article/10.3390/healthcare14060745/s1, Table S1: Descriptive characteristics of the study population and comparison between observed and imputed data (N = 516). Table S2: Non-response analysis: comparison of participants (N = 516) and non-participants (N = 563).

## Abbreviations

The following abbreviations are used in this manuscript:

PPD	Postpartum Depression
BDI-II	Beck Depression Inventory-II
EPDS	Edinburgh Postnatal Depression Scale
HPA	Hypothalamic–Pituitary–Adrenal
MICE	Multiple Imputation by Chained Equations
STAI	State-Trait Anxiety Inventory
