# Supplementary material for: Postpartum Depressive Symptoms and Their Long-Term Association with Anxiety and Depression in Women: Findings from the Rhea Study in Crete, Greece
Source: Healthcare (Basel). 2026 Mar 16;14(6):745. doi: 10.3390/healthcare14060745 (PMC13026045; doi:10.3390/healthcare14060745)
Supplement: Supplementary file 1 [file healthcare-14-00745-s001.zip › healthcare-4103967-supplementary.pdf]

Supplementary Table S1. Descriptive characteristics of the study population and comparison between observed and imputed data (N=516).

	Observed Data		20 Imputed Datasets (N=516)
	**N**	**N (%) or Mean (SD)**	**% or Mean (SE)**
**Maternal characteristics at baseline**			
Maternal age at delivery	513	30.1 (4.6)	30.0 (0.2)
Maternal origin			
Other		18 (3.5)	3.5
Greek		497 (96.5)	96.5
Maternal education in pregnancy			
Low (<9 years)		55 (10.8)	10.9
Middle (9-12 years)		258 (50.6)	50.4
High (>12 years)		197 (38.6)	38.7
Marital status in pregnancy			
Married		458 (90.7)	90.7
Other		47 (9.3)	9.3
Parity			
Primiparous		229 (46.1)	47.6
Multiparous		268 (53.9)	52.4
Working status in pregnancy			
Not working		109 (21.8)	21.7
Working		391 (78.2)	78.3
Smoking status in pregnancy			
Never smoker		321 (64.2)	64.4
Ex-smoker		87 (17.4)	17.3
Current smoker		92 (18.4)	18.4
Breastfeeding			
No		62 (12.0)	12.5
Yes		454 (88.0)	87.5
**Maternal characteristics at follow-up assessments**			
Maternal age			
At the 11-year follow-up	274	41.4 (4.3)	41.4 (0.3)
At the 15-year follow-up	462	45.5 (4.7)	45.5 (0.2)
Family income at 11 years			
Sufficient		150 (55.1)	56.3
Sufficient with problems		95 (34.9)	34.7
Insufficient		27 (9.9)	9.0
Family income at 15 years			
Sufficient		221 (47.8)	47.7
Sufficient with problems		198 (42.9)	42.8
Insufficient		43 (9.3)	9.5
Maternal working status at 11 years			
Not working		70 (25.7)	26.7
Working		202 (74.3)	73.3
Maternal working status at 15 years			
Not working		62 (13.4)	13.8
Working		400 (86.6)	86.2

Cohabitation status at 15 years			
Living with the father of the child		406 (88.1)	87.3
Other		55 (11.9)	12.7
Number of children at 15 years			
1 child		43 (9.3)	9.2
2 children		242 (52.4)	52.1
3 children		132 (28.6)	28.7
4 or more children		45 (9.7)	10.0
**Depressive symptoms**			
Postpartum period (EPDS)	516	6.3 (4.9)	6.3 (0.2)
11-year follow-up (BDI)	258	7.4 (6.5)	7.4 (0.4)
15-year follow-up (BDI)	455	7.1 (6.1)	7.1 (0.3)
**Anxiety**			
11-year follow-up (STAI)	252	42.5 (4.7)	42.5 (0.3)
15-year follow-up (STAI)	461	38.6 (8.1)	38.6 (0.4)
**Elevated depressive symptoms**			
Postpartum period (EPDS≥13)		60 (11.6)	11.6
11-year follow-up (BDI≥17)		23 (8.9)	9.3
15-year follow-up (BDI≥17)		34 (7.5)	7.4

Abbreviations: EPDS: Edinburgh Postnatal Depression Scale; BDI: Beck Depression Inventory; STAI: State-Trait Anxiety Inventory.

Supplementary Table S2. Non-response analysis: comparison of participants (N=516) and non-participants (N=563).

	Participants (N=516)	Non-participants (N=563)	
	N (%) or Mean (SD)	N (%) or Mean (SD)	p-value
**Maternal characteristics at baseline**			
Maternal age at delivery	30.1 (4.6)	29.0 (5.2)	<0.001
Maternal origin			<0.001
Other	18 (3.5)	57 (10.3)	
Greek	497 (96.5)	494 (89.7)	
Maternal education in pregnancy			<0.001
Low (<9 years)	55 (10.8)	138 (26.0)	
Middle (9-12 years)	258 (50.6)	285 (53.8)	
High (>12 years)	197 (38.6)	107 (20.2)	
Marital status in pregnancy			0.029
Married	458 (90.7)	455 (86.3)	
Other	47 (9.3)	72 (13.7)	
Parity			0.008
Primiparous	229 (46.1)	202 (38.0)	
Multiparous	268 (53.9)	330 (62.0)	
Working status in pregnancy			<0.001
Not working	109 (21.8)	174 (33.8)	
Working	391 (78.2)	341 (66.2)	
Smoking status in pregnancy			0.001
Never smoker	321 (64.2)	279 (52.8)	
Ex-smoker	87 (17.4)	118 (22.3)	
Current smoker	92 (18.4)	131 (24.8)	
Breastfeeding			0.004
No	62 (12.3)	96 (18.8)	
Yes	443 (87.7)	414 (81.2)	
**Maternal characteristics at follow-up assessments**			
Maternal age			
At the 11-year follow-up	41.4 (4.3)	38.4 (2.1)	0.220
At the 15-year follow-up	45.5 (4.7)	44.9 (4.4)	0.706
Family income at 11 years			0.845
Sufficient	150 (55.1)	1 (50.0)	
Sufficient with problems	95 (34.9)	1 (50.0)	
Insufficient	27 (9.9)	0 (0.0)	
Family income at 15 years			0.571
Sufficient	221 (47.8)	4 (66.7)	
Sufficient with problems	198 (42.9)	2 (33.3)	
Insufficient	43 (9.3)	0 (0.0)	
Maternal working status at 11 years			0.435
Not working	70 (25.7)	1 (50.0)	
Working	202 (74.3)	1 (50.0)	
Maternal working status at 15 years			0.817
Not working	62 (13.4)	1 (16.7)	
Working	400 (86.6)	5 (83.3)	

Cohabitation status at 15 years			0.368
Living with the father of the child	406 (88.1)	6 (100.0)	
Other	55 (11.9)	0 (0.0)	
Number of children at 15 years			0.841
1 child	43 (9.3)	0 (0.0)	
2 children	242 (52.4)	4 (66.7)	
3 children	132 (28.6)	2 (33.3)	
4 or more children	45 (9.7)	0 (0.0)	
**Depressive symptoms**			
Postpartum period (EPDS)	6.3 (4.9)	7.1 (5.2)	0.012
11-year follow-up (BDI)	7.4 (6.5)	9.0 (11.3)	0.737
15-year follow-up (BDI)	7.1 (6.1)	7.0 (5.7)	0.975
**Anxiety**			
11-year follow-up (STAI)	42.5 (4.7)	48.5 (4.9)	0.070
15-year follow-up (STAI)	38.6 (8.1)	48.0 (18.4)	0.104
**Elevated depressive symptoms**			
Postpartum period (EPDS≥13)	60 (11.6)	87 (15.5)	0.067
11-year follow-up (BDI≥17)	23 (8.9)	1 (50.0)	0.046
15-year follow-up (BDI≥17)	34 (7.5)	0 (0.0)	0.688

Abbreviations: EPDS: Edinburgh Postnatal Depression Scale; BDI: Beck Depression Inventory; STAI: State-Trait Anxiety Inventory.
